# [Corrigendum] Next‑generation sequencing analysis reveals that MTH‑3, a novel curcuminoid derivative, suppresses the invasion of MDA‑MB‑231 triple‑negative breast adenocarcinoma cells

**DOI:** 10.3892/or.2024.8750

**Published:** 2024-06-03

**Authors:** Yu-Jen Chiu, Fuu-Jen Tsai, Da-Tian Bau, Ling-Chu Chang, Min-Tsang Hsieh, Chi-Cheng Lu, Sheng-Chu Kuo, Jai-Sing Yang

Oncol Rep 46: 133, 2021; DOI: 10.3892/or.2021.8084

Subsequently to the publication of the article, an interested reader drew to the authors’ attention that, in Fig. 2A on p. 5, the ‘Control (24 h)’ and ‘MTH-3 (1 μM; 24 h)’ data panels contained partially overlapping data, such that they appeared to have been derived from the same original source. The authors have examined their original data, and realized that this error arose inadvertently as a consequence of having compiled this figure incorrectly.

The revised version of Fig. 2, featuring the data from one of the repeated experiments in Fig. 2A, is shown below. The revised data shown for this figure do not affect the overall conclusions reported in the paper. The authors apologize to the Editor of *Oncology Reports* and to the readership for any inconvenience caused.

## Figures and Tables

**Figure 2.**
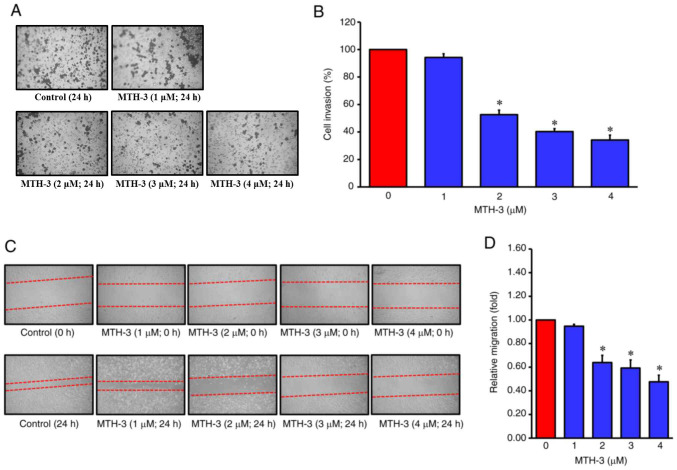
MTH-3 suppressed invasion and migration in human breast adenocarcinoma MDA-MB-231 cells. (A) The invasion ability of MDA-MB-231 cells was evaluated using a Matrigel^®^-coated invasion chamber. Following treatment with various concentrations of MTH-3 for 24 h, the invading MDA-MB-231 cells in the lower chamber were stained and subsequently counted under a light microscope (magnification, ×200). (B) Tumor cell invasion was semi-quantified. (C) The ability of migration of MDA-MB-231 cells was evaluated by wound healing assay. Following treatment with the various concentrations of MTH-3 for 24 h in serum-free Leibovitz's L-15 medium, MDA-MB-231 cells were photographed. (D) The migrated tumor cells were quantified. Data are presented as the mean ± standard deviation of three experiments. *P<0.05. MTH-3, (1E,3Z,6E)-3-hydroxy-5-oxohepta-1,3,6-triene-1,7-diyl)bis(2-methoxy-4,1-phenylene)bis(3-hydroxy-2-hydroxymethyl)-2-methyl propanoate.

